# Ureteral Ligation During Robotic-Assisted Laparoscopic Prostatectomy

**DOI:** 10.7759/cureus.66096

**Published:** 2024-08-03

**Authors:** Daniel Baetzhold, Brian Dinerman, John Rutkowski

**Affiliations:** 1 Urology, University at Buffalo Jacobs School of Medicine and Biomedical Sciences, Buffalo, USA

**Keywords:** ureteral reimplantation, prostate cancer, radical prostatectomy, robotic prostatectomy, urology surgery, ureter injury, robotic-assisted surgery

## Abstract

Robotic-assisted laparoscopic prostatectomy (RALP) is the surgical standard of care for patients with localized prostate cancer. Although uncommon, the procedure involves a potential risk of injury to adjacent anatomical structures. We report on a unique case of iatrogenic ureteral injury during RALP that required subsequent robotic-assisted laparoscopic ureteral reimplantation for definitive repair.

A 57-year-old male underwent RALP using the Da Vinci Xi system (Intuitive Surgical, Sunnyvale, CA). The procedure was unremarkable and a 20 French Foley catheter was placed with plans for removal after one week following a negative cystogram. On postoperative day two, his creatinine level elevated to 2.69 mg/dL from a baseline of 1.40 mg/dL, left-sided flank pain increased, and non-contrast CT imaging revealed moderate left proximal hydroureteronephrosis and no other abnormalities. Aside from mild nausea on postoperative day one, he had no other symptoms. An integrated stent was unable to be placed by urology at this time. Subsequently, a left percutaneous nephrostomy tube was placed under fluoroscopic guidance. After this intervention, the patient’s symptoms improved and the decision was made not to proceed with operative re-exploration at this time to attempt identification of the obstruction. Three weeks later, the patient underwent cystoscopy with attempted left retrograde ureteropyelography and left ureteroscopy due to suspected distal obstruction. This revealed complete obstruction of the intramural portion of the ureter, presumed to be secondary to suture ligation at the time of the vesicourethral anastomosis. Seven weeks postoperatively, the patient underwent robotic-assisted laparoscopic left ureteral reimplantation. Thereafter, the patient had a resolution of his left hydroureteronephrosis and acute kidney injury.

This case describes an intravesical ureteral ligation during RALP. An iatrogenic intravesical ureteral ligation has far less guiding literature than a more common ureteral transection. Additionally, ureteral transection is often identified and managed intraoperatively, while the ureteral ligation presented in this case is far less likely to be apparent during surgery. Early identification will allow for rapid reoperation to manage the injury. We hypothesize that during the vesicourethral anastomosis, the left intramural ureter was ligated. Importantly, with the use of a 3-0 V-Loc stitch for the vesicourethral anastomosis, its barbed nature would not facilitate simple surgical removal. In conclusion, when performing RALP, the depth of the bladder-sided vesicourethral anastomotic stitch should be carefully considered to avoid a similar injury.

## Introduction

Robotic-assisted laparoscopic prostatectomy (RALP) is a commonly performed procedure for localized prostate cancer. This approach is minimally invasive but has complications even with experienced surgeons [1]. Potential complications of robotic prostatectomy are vascular injury during Veress needle or trocar placement, bowel/visceral injuries, injuries due to electrocautery, pelvic nerve injury, rectal injury, and ureteric injury [2]. Complications tend to be more common in less experienced surgeons [1].

Iatrogenic injury of the ureter is relatively uncommon with a reported incidence of about 1% [2]. The ureter can be injured in the mid or distal locations during various portions of the procedure. For example, the ureter can be injured in its middle or distal portion during lymph node dissection while the distal ureter is at risk during the posterior seminal vesicle dissection. The intramural portion of the ureter can be injured during the vesicourethral anastomosis or bladder neck reconstruction. Complications are managed during the course of the procedure if recognized; however, ureteral injuries are frequently detected in the postoperative setting via changes in kidney markers and urinary function, and postoperative pain and nausea [2]. Options to temporize or definitively repair a ureteral injury include ureteral stent insertion, ureteroneocystostomy, Boari flap, ileal ureter, ureteroureterostomy, and transureteroureterostomy. We present a case of intraoperative intravesical ureteral ligation leading to hydroureteronephrosis and pain following RALP for prostate cancer.

## Case presentation

A 57-year-old male was referred for evaluation of an elevated prostate-specific antigen (PSA) of 9.7. A confirmatory PSA was performed, and his digital rectal exam was negative. His preoperative multiparametric MRI of the prostate revealed a Prostate Imaging Reporting and Data System 2 (PIRADS 2) lesion at the right apex. A subsequent transrectal prostate biopsy was performed due to concern for progression. This demonstrated a Gleason score (GS) of 4 + 4 = 8, and prostate cancer in 1/15 cores with 15/15 cores positive for prostate cancer. These findings placed him into the high-risk prostate cancer category, and his preoperative National Surgical Quality Improvement Program (NSQIP) calculation predicted a one-day hospital stay. After the risks, benefits, and alternatives were discussed with the patient, he agreed to undergo a RALP. His past medical and surgical history included hypertension, diabetes mellitus, hyperlipidemia, morbid obesity, and inguinal hernia repair with further demographic data of BMI of 48.1, weight of 280 pounds, and height of 64 inches.

The procedure was performed using a Da Vinci Xi system (Intuitive Surgical, Sunnyvale, CA) with a total of six laparoscopic ports in standard technique. A posterior approach for seminal vesicle dissection was performed. Once the prostate dissection was complete, the bladder neck was inspected, and the ureteral orifices were identified bilaterally. Following the lymph node dissection to assess for possible tumor burden, the vesicourethral anastomosis was performed. We used two 3-0 V-Loc sutures in a running fashion starting posteriorly at the five o’clock position and continuing counterclockwise and clockwise circumferentially until they were tied together at the 12 o’clock position. A 20 French Foley catheter was placed with plans for removal after one week following a negative cystogram.

On postoperative day (POD) #1, the patient reported nausea and mild postoperative pain. Serum creatinine level was 2.58 mg/dL while his baseline preoperative creatinine was 1.4 mg/dL. Typically at our institution, patients undergoing RALP are discharged on POD#1; however, the patient was observed overnight for pain control and hydration.

**Figure 1:**
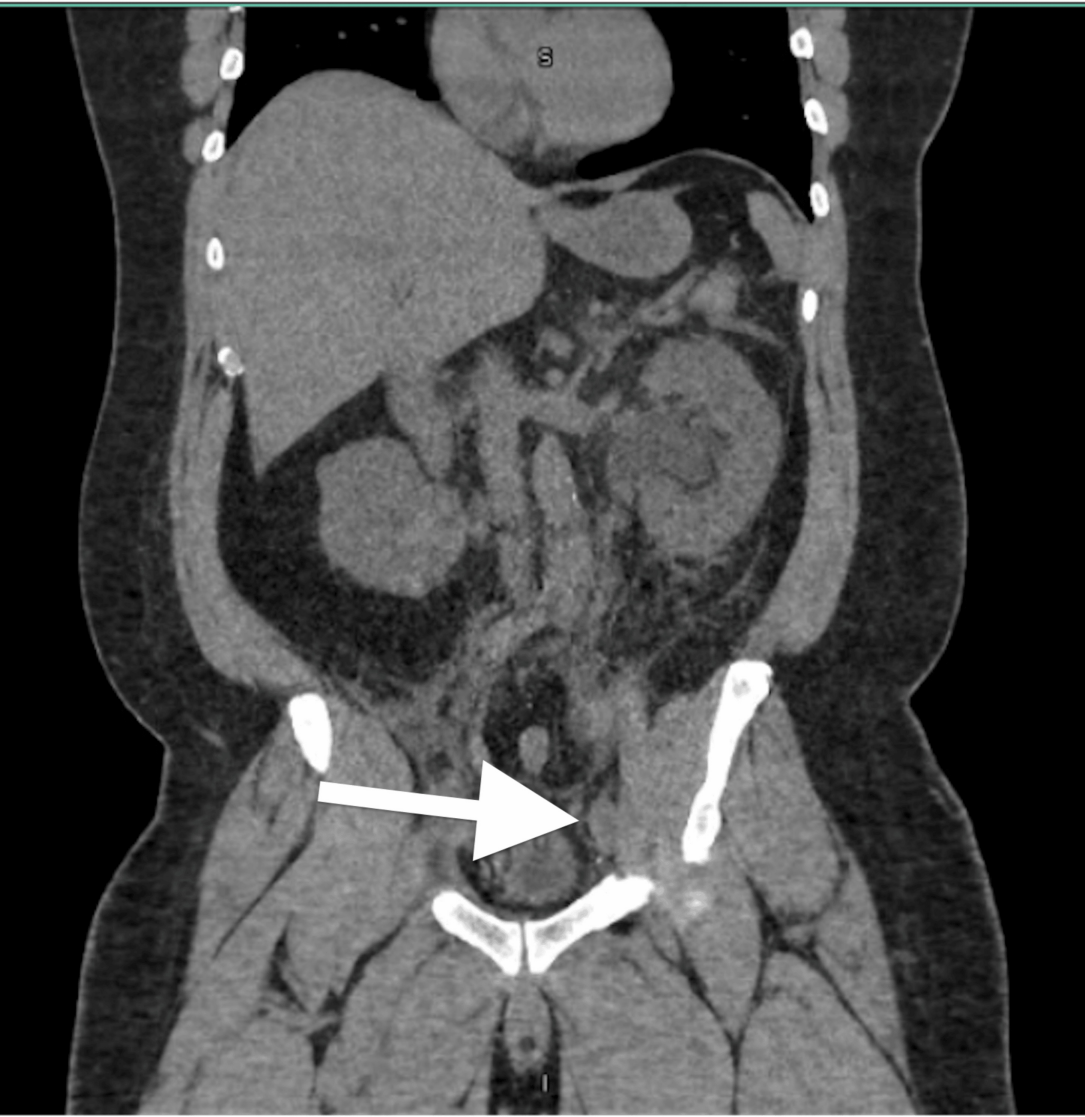
Left-sided hydroureteronephrosis on coronal non-contrast CT of the abdomen on postoperative day two.

**Figure 2:**
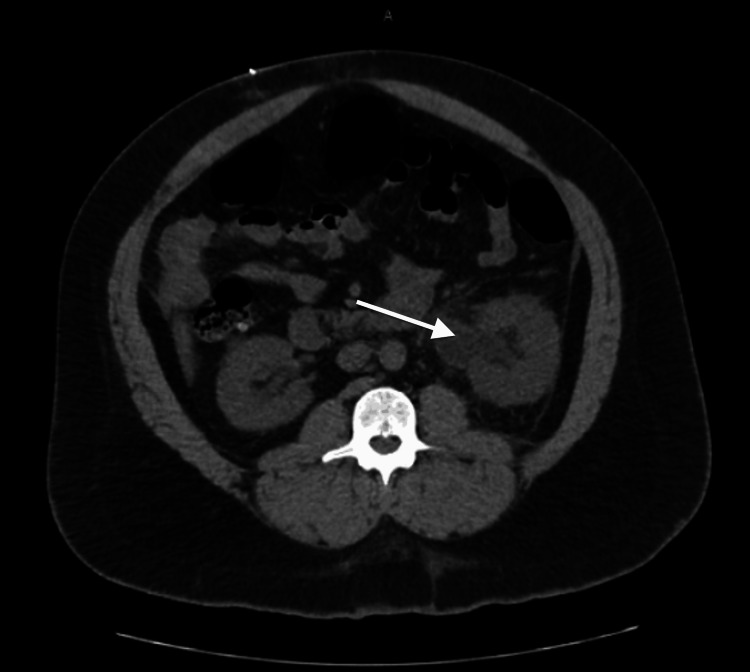
Left-sided hydronephrosis, transverse non-contrast CT of the abdomen.

On POD#2, the patient’s creatinine rose to 2.69 mg/dL, which was concerning. Concomitantly, the patient complained of moderate left flank pain. Non-contrast CT imaging of the abdomen and pelvis was performed, which revealed left hydroureteronephrosis (Figures 1, 2). A left percutaneous nephrostomy tube was placed. On POD#3, an antegrade ureteral stent was unable to be passed by interventional radiology with resistance noted in the distal ureter close to the level of the ureterovesical junction. His subsequent renal function and subjective symptoms improved after intervention with his creatinine downtrending to 1.58 mg/dL. The decision was made not to proceed with robotic re-exploration due to these clinical improvements.

Three weeks later, the patient underwent cystoscopy with attempted left retrograde pyelogram and left ureteroscopy. Although the ureteral orifice appeared normal on inspection, the pyelogram showed complete obstruction of the intramural portion of the ureter precluding placement of a guide wire. The obstruction was presumed secondary to suture ligation at the time of the vesicourethral anastomosis. Approximately two months postoperatively, the patient underwent robotic-assisted laparoscopic left ureteral reimplantation. The procedure was done robotically utilizing the same incision sites. The left ureter was identified in its mid portion at the level of the iliac vessels and mobilized distally. The dissection was rather tedious due to surgery seven weeks ago. Once an adequate length was achieved, the ureter was transected, and the distal segment was oversewn. The proximal segment was reimplanted into the bladder in the standard technique. A ureteral stent was left in place for four weeks and the nephrostomy tube was removed during the procedure. Thereafter, the patient has had resolution of his left hydroureteronephrosis and postrenal acute kidney injury. The final pathology was T3bN0, GS 3 + 4 = 7.

## Discussion

We report an intravesical ureteral injury during RALP. Radical prostatectomy is a commonly performed procedure for localized prostate cancer, and the implementation of robot-assisted techniques has become the surgical standard of care for patients with prostate cancer. The most commonly reported complications are lymphocele and urinary incontinence [1]. Bowel injury and hemorrhagic complications are also potential concerns during surgery, with a higher incidence in less experienced surgical teams [3]. Ureteral injuries occur in less than 1% of RALP and the majority are diagnosed postoperatively [2]. Notably, a posterior approach to seminal vesicle dissection may be associated with distal ureteral injury [2].

Complete and partial ureteral transections can be managed intraoperatively with a range of surgical techniques, including ureteroneocystostomy, ureteroureterostomy, primary repair, and most infrequently, transureteroureterostomy [4,5]. Injury to the ureteral orifice is also possible during robotic prostatectomy, especially if bladder neck reconstruction is required after the removal of larger prostate glands. In one reported case, a surgeon lacerated the left ureteral orifice during bladder neck dissection in a patient with a prominent median lobe. A ureteral stent was inserted to maintain the patency of the ureteral orifice [6].

Like other ureteral injuries sustained during RALP, this problem was not identified until POD#2. This case describes a ureteral injury poorly defined elsewhere in the literature. An iatrogenic intravesical ureteral ligation has far less guiding literature than a ureteral transection. Surgical timing should also play a role in surgical management. Early identification in the postoperative period allows for rapid reoperation to repair the injury. However, if the injury is identified more than one week postoperatively, it is prudent for the surgeon to delay definitive repair in the order of months after injury to minimize postoperative inflammation and urine extravasation interference. In this case, the clinical improvement of the patient after the initial operation led to delayed diagnosis, and thus delayed repair to allow for postoperative healing. We hypothesize that during the vesicourethral anastomosis, the left intramural ureter was ligated. Importantly, with the use of a 3-0 V-Loc stitch for the vesicourethral anastomosis, its barbed nature would not facilitate simple postoperative surgical removal. In conclusion, when performing RALP, the depth of the bladder-sided vesicourethral anastomotic stitch should be carefully considered to avoid a similar injury.

## Conclusions

This case report identifies an instance of iatrogenic hydroureteronephrosis secondary to ureteral ligation during robot-assisted radical prostatectomy. More research is necessary to identify risk factors for surgical errors as well as standardize care to treat uncommon injuries caused by these instances. This case highlights the importance of the anastomotic stitch in the vesicourethral junction to avoid a similar injury.
